# Trehalose Reduces the Secreted Beta-Amyloid Levels in Primary Neurons Independently of Autophagy Induction

**DOI:** 10.3390/metabo11070421

**Published:** 2021-06-26

**Authors:** Irene Benito-Cuesta, Lara Ordoñez-Gutierrez, Francisco Wandosell

**Affiliations:** 1 Centro de Biología Molecular “Severo Ochoa” (CSIC-UAM), Universidad Autónoma de Madrid, 28049 Madrid, Spain; lordoniez@cbm.csic.es; 2 Centro de Investigación Biomédica en Red de Enfermedades Neurodegenerativas (CIBERNED), 28049 Madrid, Spain

**Keywords:** Alzheimer, amyloid accumulation, autophagy, bafilomycin A1, cultured cerebellar granule neuron, SH-SY5Y, trehalose

## Abstract

The disaccharide trehalose was described as possessing relevant neuroprotective properties as an mTORC1-independent inducer of autophagy, with the ability to protect cellular membranes and denaturation, resulting from desiccation, and preventing the cellular accumulation of protein aggregates. These properties make trehalose an interesting therapeutic candidate against proteinopathies such as Alzheimer’s disease (AD), which is characterized by deposits of aggregated amyloid-beta (Aβ) and hyperphosphorylated tau. In this study, we observed that trehalose was able to induce autophagy in neurons only in the short-term, whereas long-term treatment with trehalose provoked a relevant anti-amyloidogenic effect in neurons from an AD mouse model that was not mediated by autophagy. Trehalose treatment reduced secreted Aβ levels in a manner unrelated to its intracellular accumulation or its elimination through endocytosis or enzymatic degradation. Moreover, the levels of Aβ precursor protein (APP) and beta-secretase (BACE1) remained unaltered, as well as the proper acidic condition of the endo-lysosome system. Instead, our results support that the neuroprotective effect of trehalose was mediated by a reduced colocalization of APP and BACE1 in the cell, and, therefore, a lower amyloidogenic processing of APP. This observation illustrates that the determination of the mechanism, or mechanisms, that associate APP and BACE is a relevant therapeutic target to investigate.

## 1. Introduction

Some of the properties described for trehalose make this sugar an interesting therapeutic candidate against abnormal protein aggregation observed in age-related neurodegenerative diseases, including Alzheimer’s disease (AD). Trehalose is composed of two glucose molecules linked by an α,α (1-1) glycosidic bond (α-D-glucopyranosyl-(1-1)-α-D-glucopyranoside) [1]. As a nonreducing disaccharide, it is highly resistant to acid hydrolysis and glucosidases in trehalose-free organisms, which include mammalian cells. This sugar can be synthetized by organisms ranging from bacteria, yeast, and fungi, to insects, invertebrates, and plants. Trehalose has shown relevant neuroprotective functions as a potent mTORC1-independent autophagic inductor [2,3,4,5]. However, there is no general consensus regarding the mechanism of the induction of autophagy [6,7,8,9,10,11,12]. Moreover, this disaccharide is considered to protect cellular membranes, preventing protein denaturation by direct unions, thus protecting the cell against environmental stresses [1,13]. These properties make trehalose an interesting therapeutic approach against neurodegenerative proteinopathies such as Alzheimer’s disease (AD) [10]. As post-mitotic cells, neurons are especially vulnerable since they cannot dilute harmful protein aggregates or dysfunctional organelles through cellular division [14]. In fact, dysfunction in degradation systems (e.g., autophagy) has been associated with the abnormal protein aggregation observed in age-related neurodegenerative diseases including AD [15,16,17,18]. AD is characterized by deposits of aggregated amyloid-beta (Aβ) and hyperphosphorylated tau proteins. The mature Aβ peptide is generated by the sequential cleavage of Aβ precursor protein (APP) by beta- and gamma-secretases, a process known as the amyloidogenic pathway. The main beta-secretase (BACE1) processes APP mostly after its internalization in late endosomes and lysosomes, since BACE1 needs an acidic pH for optimal endoproteolytic activity [19,20]. An accumulation of Aβ-containing autophagic vesicles, whose maturation and degradation remain impaired, was described in AD [10]. Increasing evidence supports the promotion of autophagy as a therapeutic strategy for the amelioration of such AD pathology [21,22], and trehalose has been proposed as a therapeutic mTOR-independent autophagy inducer in several neurodegenerative diseases [2,5,10]. Autophagy is a catabolic process by which cells degrade unnecessary or dysfunctional components through the action of lysosomes [23,24]. From the three main subtypes (for a review see: [25,26]), macroautophagy (hereafter referred to simply as autophagy) is highly characterized in the nervous system [22]; and consists of the sequestration of a portion of the cytoplasm in a double-membraned autophagic vesicle or autophagosome (AV), for its subsequent fusion with the lysosome and degradation of its content. Briefly, the ULK1/2 complex activates the phosphatidylinositol 3-kinase class III (PI3KCIII) complex, leading to the primary nucleation of membrane and recruitment of ATG proteins. During membrane elongation, phosphatidylethanolamine attaches to the cytosolic form of microtubule-associated protein 1 light chain 3 (LC3I) to become the specific autophagosome membrane-associated form LC3II [27,28,29]. Autophagic adaptors, such as p62/SQSTM1 (Sequestosome 1) or NBR1 (the neighbor of the BRCA1 gene), recognize both cytoplasmic substrates and the LC3II protein [30,31]. Finally, the autophagosome fuses its outer membrane with a lysosome, forming a single membrane autophagolysosome, to degrade its contents and restore the lysosome.

In the present study, we analyzed the ability of trehalose to induce autophagy in neurons and its effect on the pathological accumulation of Aβ in AD. For this purpose, we employed a double-transgenic APP/PS1 mouse model, which accumulated human Aβ peptides. We observed that trehalose was able to induce autophagy in neurons only in the short-term. Long-term treatments with trehalose provoked a modification in the endomembrane system, segregation of APP and BACE1, preventing the amyloidogenic pathway, and, consequently, a therapeutic reduction of Aβ production.

## 2. Results

### 2.1. Trehalose Only Increases the Neuronal Autophagic Flux in the Short Term

As a preliminary approach, we used the neuroblastoma cell line SH-SY5Y to determine the trehalose exposure time needed to generate an effect. We analyzed autophagic markers at different exposure times (30 min to 48 h) to 100 mM trehalose in SH-SY5Y (Supplementary Materials Figure S1A). We observed a time-dependent increase of the autophagosome marker LC3-II, but also higher levels of the autophagic receptors SQSTM1 and NBR1 after 24 h and 48 h of treatment. This effect differs from that observed with the autophagy inducer rapamycin (Figure S1B, and previously reported in [21]), but resembles, to a lesser extent, that observed with the v-ATPase inhibitor BafA1, which blocks lysosomal degradation and therefore provokes the accumulation of non-degraded autophagosomes, shown as the increase of autophagic markers LC3-II, NBR1, and SQSTM1 (Figure S1B). Interestingly, trehalose decreased the phosphorylation levels of RPS6 within the first hour of treatment, which gradually recovered to control levels over the next hours, contrary to the progressive decrease observed with BafA1 or the sustained inhibition of rapamycin (Figure S1A,B).

Next, we confirmed the long-term effects of trehalose in primary cerebellar granule neurons (CGNs), obtained from P5-P7 mice treated with trehalose (50 mM or 100 mM) for 24 or 48 h (Figure S1C). Similarly, we observed a time- and concentration-dependent increase of LC3-II levels without any effect on mTORC1 signaling (inferred by its phosphorylation targets RPS6KB1 or RPS6) or cellular viability according to cleaved CASP3 levels (Figure S1C). These preliminary data suggest a different effect of trehalose on the autophagic status of the cell, depending on the duration of the treatment.

Increased LC3II levels could be a consequence of autophagy induction or due to a blockage of the lysosomal degradation. Therefore, in order to study the autophagic degradation efficiency, we analyzed the autophagic flux, which refers to the dynamic process of autophagosome generation and degradation by fusion with the lysosome [14,27]. To estimate the autophagic flux, we used BafA1 to block the degradation by the lysosome, therefore leading to the accumulation of autophagic markers. This accumulation of autophagic markers with BafA1 was higher when autophagy was induced, as observed in the presence of rapamycin when compared to control conditions.

**Figure 1:**
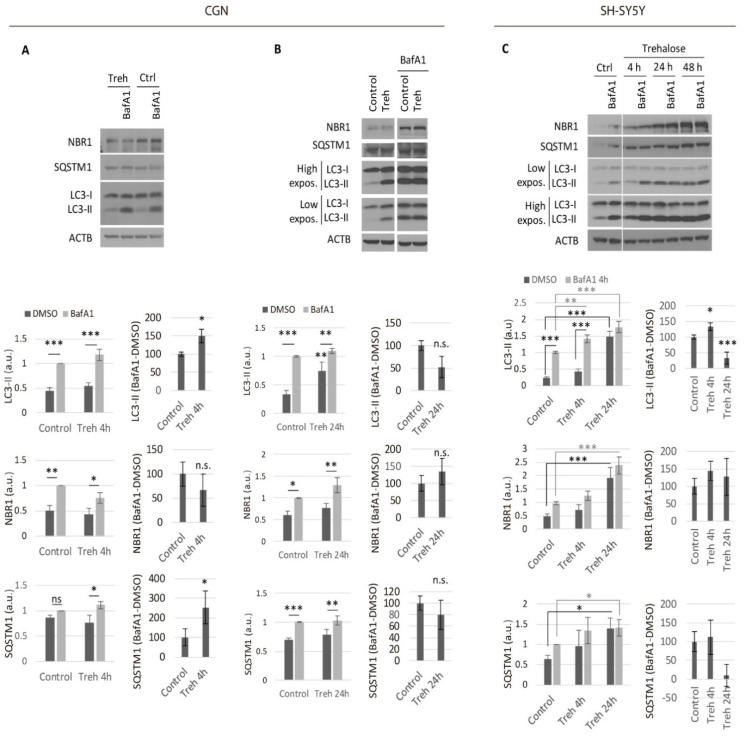
Trehalose only increased the neuronal autophagic flux in the short term. Autophagic flux analysis by addition of 100 nM BafA1(or DMSO as vehicle) during the last 4 h of treatment with 100 mM of Treh for 4 h (**A**) or 24 h (**B**) in CGN cultures; and (**C**) autophagic flux analysis by addition of 100 nM of BafA1 during the last 4 h of treatment with 100 mM of Treh for 4 h, 24 h or 48 h in SH-SY5Y cultures. Two-way ANOVA was performed for NBR1, SQSTM1, and LC3-II levels. The quantitative data, BafA1 versus the control, were normalized and represented as 100 relative units. Student’s t-test was performed for comparisons of NBR1, SQSTM1, and LC3-II levels in the presence-absence of BafA1 (BafA1-DMSO). (*n* ≥ 3; * *p* ≤ 0.05; ** *p* ≤ 0.01; and *** *p* ≤ 0.001). Bars represent mean ± SEM. NBR1, SQSTM1, and LC3-II were measured to determine the autophagic status and ACTB as an internal control. n.s.: not significant.

Thus, to estimate the status of autophagic flux after short- (4 h) and long-term (24 h) treatment of CGNs (Figure 1A,B) or SH-SY5Y (Figure 1C) with 100 mM trehalose, we compared levels of the autophagy-specific markers in the presence and absence of 100 nM BafA1, added 4 h before the cell harvest. Both neuronal systems CGN (Figure 1A) and SH-SY5Y (Figure 1C) showed an increased autophagic flux after 4 h of treatment with trehalose, as shown by the significantly higher levels of LC3-II in the presence of BafA1 compared to the controls. A higher accumulation of SQSTM1 in the presence of BafA1 with trehalose confirmed this effect in CGNs (Figure 1A), although no significant differences were observed in SH-SY5Y (Figure 1C) or NBR1 levels (Figure 1A,C). However, long-term treatment (24 h) with trehalose did not maintain the observed higher autophagic flux in CGNs (Figure 1B) or SH-SY5Y (Figure 1C). In fact, there was a significant decrease of autophagic-flux in SH-SY5Y after long-term treatment with trehalose (Figure 1C), although this effect was not statistically significant in primary neuronal cultures (Figure 1B).

### 2.2. Trehalose Reduces Aβ40 Secretion in Primary Neurons Isolated from APP/PSEN1 Mice in an Autophagy Independent Fashion

**Figure 2:**
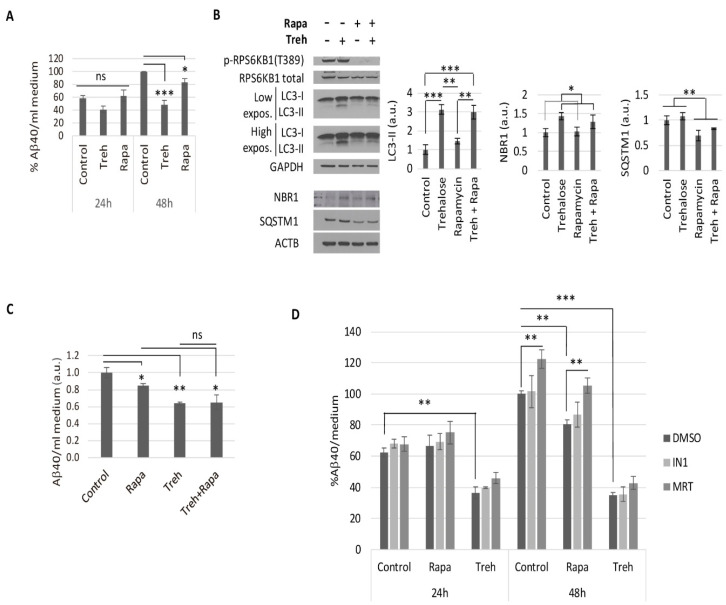
The reduction of secreted Aβ40 in neurons by trehalose was not mediated by autophagy. The (**A**) APP/PSEN1 CGN cultures were treated with 100 mM of trehalose (Treh) or 200 nM of rapamycin (Rapa) for 24 or 48 h, and Aβ40 levels were detected in culture media by ELISA. Two-way ANOVA was performed (*n* ≥ 4). The (**B**,**C**) APP/PSEN1 CGN cultures were treated with 100 mM of Treh or 200 nM of Rapa for 48 h. The (**B**) cell extracts were analyzed by western blot. NBR1, SQSTM1, and LC3-II were measured to determine the autophagic status, with p-RPS6KB1 T389 as an indicator of MTORC1 activity, and ACTB and GAPDH as internal controls. Two-way ANOVA was performed (*n* = 3). The (**C**) Aβ40 levels in the culture media were detected by ELISA. Two-way ANOVA was performed (*n* = 3). The (**D**) Aβ40 levels in culture media were determined by ELISA after 24 or 48 h of treatment with 100 mM of Treh in the presence or absence of the inhibitors of autophagosome formation PIK3C3/VPS34-inhibitor 1 (IN1) or MRT68921 (MRT) (24 h: 0.25 μM of IN1 or 0.5 μM of MRT; 48 h: 0.1 μM of IN1 or 0.1 μM of MRT). Two-way ANOVA was performed for 24 and 48 h of treatment (*n* ≥ 3). (ns: non-significant; * *p* ≤ 0.05; ** *p* ≤ 0.01; and *** *p* ≤ 0.001). Bars represent mean ± SEM.

Despite trehalose inducing autophagy only in the short-term, we wondered if it would be able to modify the secretion of amyloid-beta in neurons. We treated CGNs obtained from APP/PSEN1 mice with trehalose for 24 or 48 h, and harvested the media to measure secreted Aβ40 levels by ELISA (Figure 2A). Our data showed a drastic 50% reduction of secreted Aβ40 after 48 h of trehalose treatment, much higher than that due to the induction of autophagy by a 200 nM rapamycin treatment (Figure 2A).

We studied the relevant decrease of secreted amyloid levels by trehalose to determine if it was mediated by autophagy. First, we analyzed if trehalose affected the autophagy-mediated degradation by rapamycin after 48 h. By western blot of CGN extracts, we observed that the lower levels of SQSTM1, due to the higher degradation rate with rapamycin, were not affected by trehalose (Figure 2B). Conversely, the higher levels of LC3-II and NBR1 seen during trehalose treatment were not significantly reverted by rapamycin (Figure 2B). These data confirmed that long-term treatment with trehalose did not block the autophagy-lysosome degradation process. We did not observe any additive effect when analyzing the secreted Aβ40 levels by ELISA (Figure 2C), suggesting that the anti-amyloidogenic effect of trehalose was not mediated by autophagy.

To confirm that the anti-amyloidogenic effect of trehalose was independent of autophagy, we prevented the autophagy process by blocking the initial formation of autophagosomes. For this purpose, we used PIK3C3/VPS34-inhibitor 1 (IN1), which specifically inhibits the PIK3C3 subunit of class III PtdIns3K complex without affecting other PIK3s [32,33], and MRT68921 (MRT), a potent specific inhibitor of the ULK complex kinases ULK1/2 [34]. We treated CGNs for 48 h with or without trehalose and in the presence or absence of IN1 or MRT inhibitors (Figure 2D and Figure S2). Western blot analysis confirmed that both inhibitors, IN1 and MRT, were able to prevent autophagosome formation [21] and therefore block the increase of LC3-II levels observed with trehalose (Figure S2). The prevention of autophagosome formation by IN1 or MRT did not modify the decreased secreted Aβ40 levels by trehalose, in contrast to the higher amyloid levels observed when preventing basal or rapamycin-induced autophagy with MRT (Figure 2D). These data confirm that trehalose induced a reduction of secreted Aβ40 levels by neurons in an autophagy-independent fashion.

### 2.3. Lowered Secreted Levels of Aβ40 by Trehalose Are Not Due to Its Intracellular Storage or to a Higher Endocytosis Rate

**Figure 3:**
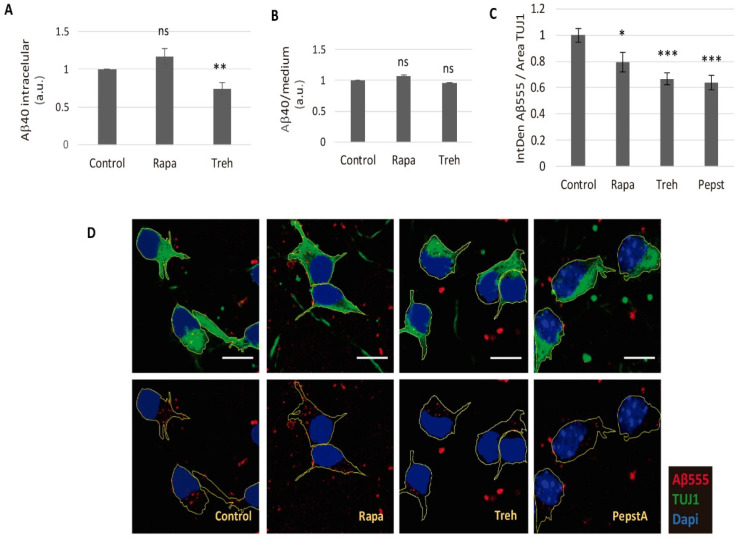
Trehalose did not block the secretion or endocytosis of amyloid beta peptide. The (**A**) APP/PS1 CGN cultures were treated with 100 mM of trehalose (Treh) or 200 nM of rapamycin (Rapa) and intracellular Aβ40 levels in cell extracts were detected by ELISA. Student’s t-test was performed (*n* = 6). The (**B**) CGN cultures from wild-type mice were treated for 48 h with 100 mM of Treh or 200 nM of Rapa in conditioned media from cultured APP/PS1 CGN. The remaining Aβ40 levels in culture media were detected by ELISA. One-way ANOVA was performed (*n* = 3). The (**C**,**D**) CGN cultures from wild-type mice were treated for 4 h with 100 mM of Treh, 200 nM -of Rapa or 10 µg/mL of pepstatin A in the presence of 2 µg/mL HiLyte™ Fluor 555 labeled-Aβ (1-40) (Aβ555), and the internalized Aβ555 was measured by confocal microscopy. The (**C**) quantification of Aβ555 intensity in relation to the neuronal area; and (**D**) representative images: Aβ555 (red), TUJ1 (green), and dapi (blue). Forty-five neurons were quantified per experimental condition. (ns: non-significant; * *p* ≤ 0.05; ** *p* ≤ 0.01; and *** *p* ≤ 0.001). Bars represent mean ± SEM.

To determine if the lower secreted Aβ40 levels after trehalose treatment were due to a deficiency in the secretion process, we studied whether the peptide was retained inside the cell. We treated cultured CGNs, obtained from APP/PSEN1 mice, with trehalose for 48 h, and then harvested the cells to measure the intracellular Aβ40 levels by ELISA (Figure 3A). Trehalose did not generate an increase of the intracellular Aβ40 levels, but a decrease was statistically significant, which rules out storage inside the cell.

An alternative hypothesis, that trehalose accelerates the endocytosis-mediated degradation of Aβ40 after its secretion, was considered. To test this, we treated cultured CGNs from WT mice with trehalose or rapamycin in an Aβ-rich conditioned media, obtained from APP/PS1 CGNs, for 48 h. A comparison of the remaining extracellular (Figure 3B) or intracellular (data not shown) Aβ40 levels by ELISA did not show any significant difference. As a complementary approach, we treated cultured CGNs from WT mice with 100 mM of trehalose in the presence of HiLyte™ Fluor 555 labeled-Aβ (1-40) (Aβ555) for 4 h, and the internalized Aβ was measured by immunofluorescence (Figure 3C,D). We used 10 μg/mL pepstatin A as a negative control, as it was described as able to block endocytosis [35]. Both trehalose and rapamycin showed a significant decrease in the internalization of Aβ (Figure 3C,D). Therefore, trehalose did not diminish the levels of secreted Aβ through enhancement of its endocytosis.

### 2.4. Trehalose Diminishes the Amyloidogenic Processing of APP

**Figure 4:**
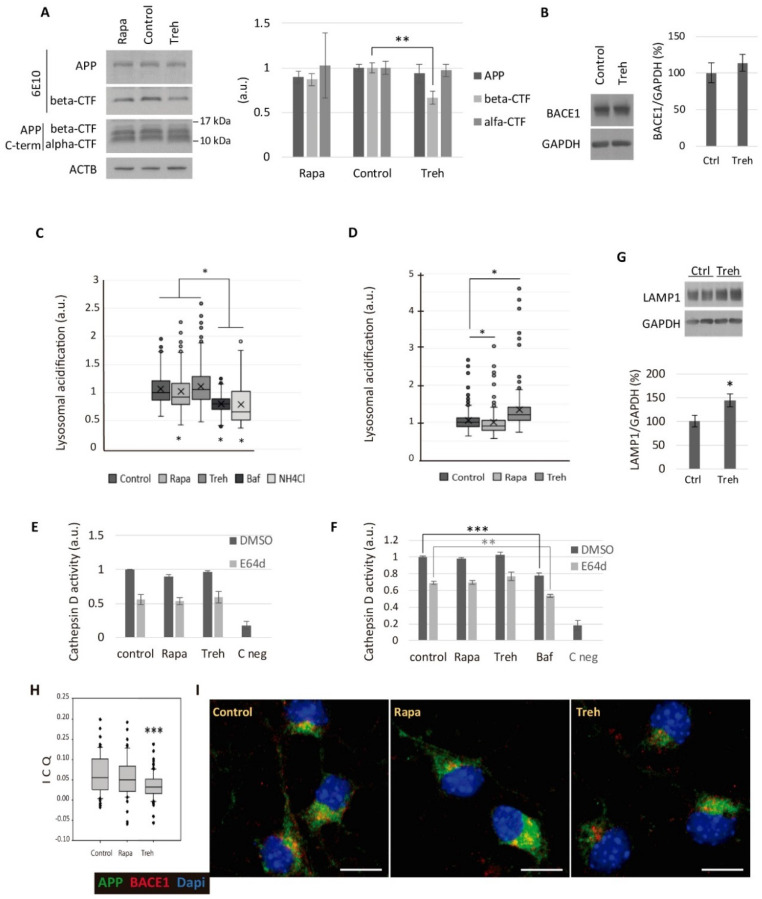
Trehalose diminished the amyloidogenic processing of APP in CGN cultures. The (**A**) CGN cultures were treated for 24 h with 100 mM of Treh or 200 nM of rapamycin (Rapa). Western blot analysis of APP, beta-CTF (measured with 6E10 antibody), and alpha-CTF (APP C-term antibody) was conducted. Student’s t-test was performed. The (**B**) western blot analysis of BACE1 levels in CGN cultures treated with 100 mM of trehalose. The (**C**,**D**) CGN cultures were treated for 4 h with 200 nM of rapamycin, 100 mM of trehalose, 100 nM of bafilomycin A1 (Baf) or 15 mM of NH4Cl (**C**); or for 24 h with 200 nM of rapamycin or 100 mM of trehalose (**D**). During the last hour of treatment, CGNs were incubated with the acidotropic probe LysoSensor™ Green DND-189 to determine the acidification level of the endo-lysosomal system with an inverted fluorescence microscope. One-way ANOVA on ranks was performed. The (**E**,**F**) cathepsin D activity assay of CGN cultures treated for 24 h with 200 nM of rapamycin or 100 mM of trehalose (**E**); or for 4 h with 200 nM of rapamycin, 100 mM of trehalose or 100 nM of bafilomycin A1 (**F**). C neg: blank solution. Two-way ANOVAs were performed. The (**G**) western blot analysis of LAMP1 levels after treating CGN cultures with 100 mM trehalose for 24 h. Student’s t-test was performed. The (**H**,**I**) colocalization analysis of APP (22C11, green) and BACE1 (red) in CGN cultures treated for 24 h with 200 nM of rapamycin or 100 mM of trehalose. The intensity correlation analysis (ICA) algorithm was employed to determine the intensity correlation quotient (ICQ) in ImageJ software. Student’s t-test was performed. Nuclei were stained with DAPI. Scale bar: 10 μm. Seventy neurons were quantified per experimental condition. (* *p* ≤ 0.05; ** *p* ≤ 0.01; and *** *p* ≤ 0.001). Bars represent mean ± SEM. Box plots represent 10th, 25th, 50th, 75th, and 90th percentiles as boxes and error bars, while outliers are represented as dots.

Since the elimination of the amyloid peptide through autophagy, endocytosis, or secretion was not promoted, we studied whether trehalose was modifying the amyloidogenic processing of APP. We treated cultured CGNs obtained from APP/PSEN1 mice with trehalose or rapamycin for 24 h to analyze the pro- and anti-amyloidogenic processing of APP by western blot (Figure 4A). We did not observe any modification of the total APP or alpha-CTF fragment levels derived from the non-amyloidogenic pathway. The levels of alpha-secretase ADAM10 did not change either (data not shown). Interestingly, trehalose generated decreased levels of beta-CTF (Figure 4A), the fragment derived from the amyloidogenic processing of APP by BACE1. However, we did not observe a reduction in the levels of the β-secretase BACE1 in the presence of trehalose (Figure 4B).

Since BACE1 needs an acidic pH for an optimal endoproteolytic activity [20], we investigated whether trehalose was modifying the endo-lysosomal activity. We treated CGN cultures with trehalose and measured the acidification level of the endo-lysosomal system with the acidotropic probe LysoSensor™ Green DND-189, which accumulates in acidic organelles and exhibits a pH-dependent increase in fluorescence intensity upon acidification. Contrary to 100 nM of BafA1 or 15 mM of NH4Cl for 4 h (Figure 4C), treatment with 100 mM of trehalose for 4 or 24 h did not inhibit the acidification of the endo-lysosomal system (Figure 4C,D).

To confirm that trehalose was not hampering the pH-dependent proteolysis, we performed an enzyme activity assay of the aspartic protease cathepsin D, which constitutes a major component of lysosomes, but also has beta-secretase activity [36]. We treated CGN cultures with trehalose for 24 h or 4 h, and cell extracts were incubated with a fluorogenic cathepsin D and E substrate. We added the inhibitor E64d to prevent the additional activation of cathepsin D by cysteine proteases [37,38]. The analogous decrease of the signal with E64d indicated that there were no differences in regulation by cysteine proteases between treatments (Figure 4E,F). Whereas BafA1 led to a significant reduction of cathepsin D activity in accordance with its lysosomal basification effect, neither trehalose nor rapamycin modified its activity at 24 or 4 h (Figure 4E,F).

Despite the fact that lysosomal activity was not diminished by trehalose, we did observe a significant increase in the lysosomal marker LAMP1 (Figure 4G). By transmission electron microscopy of cultured CGNs, we observed alterations in the endomembrane system provoked by trehalose: large vesicles, in accordance to the higher LAMP1 levels, and wider cisternae of the Golgi apparatus (Figure S3). Therefore, we questioned whether trehalose could be reducing the colocalization of APP and BACE1, and, consequently, the generation of Aβ peptide. We did not observe differences in colocalization of APP-LAMP1 (Figure S4A) or BACE1-LAMP1 (Figure S4B). However, there was a significant reduction of APP-BACE1 colocalization (Figure 4H,I).

To reinforce this data, we treated CGN cultures with trehalose and performed sucrose gradients to analyze several membrane compartments. The fractions obtained were analyzed by western blot using antibodies against lysosome-endosome compartments (LAMP1-Rab7), APP and BACE, with ACTB and GAPDH as controls. The ratio APP/BACE1 in each fraction showed a different redistribution of both proteins in the cellular compartments when treating the cells with trehalose as compared to control conditions (Figure S5). This data was consistent with the modification observed in the composition of the lysosomal compartment (LAMP1), which showed a switch to lighter fractions (Figure S5). BACE1 also showed a trend to be in a lighter fraction, and the redistribution of APP *versus* LAMP1 fractions showed a statistically significant difference (Figure S5).

Altogether, these data support that trehalose modified the endomembrane system, and therefore the trafficking of APP and BACE1 in neurons, preventing the pro-amyloidogenic processing of APP and reducing the secretion of Aβ.

## 3. Discussion

Trehalose has been described as a potent inducer of autophagy with neuroprotective effects and, although its molecular mechanism remains elusive [1,10], some hypotheses do exist. For instance, it was proposed that it acts as a “water replacement,” taking the place of water in the desiccated organism by hydrogen bonding interactions with polar groups on membrane lipids and proteins [39].

Our results show that trehalose has an anti-amyloidogenic effect in neurons of the AD mouse model APP/PS1. However, trehalose was able to induce autophagy only in the short-term (4 h of treatment), whereas this pro-autophagic effect was completely lost in the long-term in neurons. Therefore, the huge decrease of Aβ levels in the presence of trehalose was not mediated by the anti-amyloidogenic effect of autophagy in CGN [5]. The long-term treatment with trehalose did not reverse the higher autophagic-dependent degradation of SQSTM1 by the autophagy inducer rapamycin (Figure 2), nor did it affect the acidification or enzymatic activity of the lysosome (Figure 4), ruling out a blockage of the lysosomal degradation. Therefore, long-term treatment with trehalose resulted in higher levels of LC3-II and LAMP1 without affecting the degradation capacity of the autophagy-lysosome pathway. This time-dependent effect of trehalose may explain the contradictory results described in the literature about its ability to induce autophagy [11]. Tien et al. described an altered subcellular distribution of APP in H4 neuroglioma cell line, independent of autophagy induction [40]. However, they described an inhibition of lysosomal degradation and activation of cathepsin D by trehalose, resulting in increased APP and CTF levels in H4 neuroglioma cells [39]. In this study, we did not observe a decreased degradation of APP nor impaired lysosomal activity (according to acidification and enzymatic assays). Contrary to the general accumulation of CTFs with trehalose in cell lines, as described by Tien et al., we did not appreciate modifications in the levels of alpha-CTFs, but we observed a specific decrease in the levels of beta-CTF in primary neurons, supporting a reduction of the pro-amyloidogenic processing of APP by BACE1 [40]. Rusmini et al. observed in an immortalized motoneuronal cell line that long-term treatment with trehalose enhanced the expression of SQSTM1 and LC3, inducing autophagy, but also promoting the expression of the autophagy repressor ZKSCAN3 [12]. Accordingly, we saw increased levels of SQSTM1 (in SH-SY5Y) and LC3-II (in CGNs and SH-SY5Y) after 24 h of treatment with trehalose; thus, we hypothesized that expression of the repressor ZKSCAN3 would explain why induction of autophagy is lost after long-term treatments [12]. However, according to the LysoSensor™ and cathepsin D activity assays at both 4 and 24 h, we did not see the lysosomal impairment after short-term trehalose treatment described by Rusmini et al., although we did observe an enlargement of lysosomes [12].

Regarding autophagy induction, different potential molecular mechanisms were described for trehalose. DeBosch et al. observed internalization of trehalose through SLC2A8 (GLUT8), inhibiting glucose transport and enhancing autophagy by AMPK activation [6,8]. The activation of TFEB by trehalose was described as mediated by the inhibition of AKT [9] and activation of PPP3CB (protein phosphatase 3, catalytic subunit, beta isoform) [12]. However, we did not observe any modification of the phosphorylation levels of AKT, AMPK or its targets ACAC1 and ULK1 (data not shown).

According to our results, the long-term anti-amyloidogenic effect of trehalose was not mediated by autophagy, as suggested by autophagy markers and confirmed by the prevention of autophagosome formation using the inhibitors MRT or IN1, which did not reverse the trehalose effect on Aβ levels. We also demonstrated that the reduced extracellular Aβ levels after trehalose treatment were unrelated to intracellular accumulation, endocytosis, or degradation by extra- or intracellular enzymes.

Furthermore, trehalose reduced Aβ levels without altering APP or BACE1 levels. Since the beta-secretase BACE1 requires an acidic pH for optimal endoproteolytic activity [20], we analyzed the lysosomal compartment. Trehalose did not impair the acidic pH of lysosomes or cathepsin D activity, allowing us to rule out a dysfunctional beta-secretase activity. Although we did not observe differences in the colocalization of APP-LAMP1 or BACE1-LAMP1, trehalose treatment led to enlarged lysosomes with higher levels of LAMP1, and sucrose gradient experiments showed a shift of BACE1 and LAMP1 to a lighter fraction, whereas APP redistributed to denser cellular fractions. Accordingly, immunofluorescence experiments showed a reduced colocalization of APP and BACE1 when treating the neurons with trehalose.

The reduced cellular colocalization of APP and BACE1 with trehalose, together with the altered endomembrane system, supported a segregated trafficking of both proteins, preventing this initial amyloidogenic cleavage and therefore leading to the huge decrease of Aβ levels observed.

APP and BACE1 interact in both biosynthetic and endocytic compartments of neurons. In particular, upon activation, APP is routed into BACE1-positive recycling endosomes via an uncharacterized clathrin-dependent mechanism [41,42]. Unlike sucrose, trehalose is not degraded by mammal cells and was described as able to cross the blood-brain barrier [43]. Trehalose analogs that are available could be used to avoid the intestinal degradation when orally taken [44]. Therefore, trehalose constitutes an interesting therapeutic approach against AD, although more research is needed to determine its in vivo effects and molecular mechanism.

In summary, here we showed an autophagy-independent anti-amyloidogenic effect of trehalose in neurons of an AD mouse model. Our data support that this anti-amyloidogenic effect was not due to intracellular retention or extracellular degradation.

This trehalose-dependent amyloidogenic reduction occurred without altering the APP or BACE1 levels, or the acidic conditions optimal for the β-secretase activity. Instead, trehalose treatment reduced APP-BACE1 cellular colocalization. With all these observations, it is may be proposed that trehalose impairs the colocalization of BACE1 and APP, thus disrupting the internalization process that initiates the amyloidogenic cascade.

## 4. Materials and Methods

### 4.1. Animal Handling

We used the double-transgenic mouse strain B6.Cg-Tg (APPSwe, PSEN1dE9) 85Dbo/J, which overexpresses the human genes APP (amyloid beta precursor protein), with the Swedish mutation and exon-9-deleted PSEN1 (presenilin 1; Jackson Laboratory, Bar Harbor; ME, USA, stock no. 005864), hereafter referred to as APP/PSEN1 [45,46]. Each mouse’s genotype was confirmed by PCR of DNA isolated from tail biopsies [47]. All animal care and handling strictly followed current Spanish guidelines and legislation, and those of the European Commission (directive 2010/63/EU). The use of wild-type and transgenic animals was an absolute requirement for this project. All the procedures for use and management of the transgenic colony were approved by the Spanish Research Council (CEEA-CBMSO-33/307), the Community of Madrid (PROEX 341/15), recently extended by 5 years, the Spanish Research Council (CEEA-CBMSO-23/307.1), and the Community of Madrid (Ref.: PROEX 069.7/21). All transgenic mice and non-transgenic littermates were group-housed in standard cages with fiber bedding, under a 12 h/12 h light and dark cycle. Mice were housed under constant temperature (22 ± 2 °C) and humidity (50 ± 5%) in a specific-pathogen-free animal facility.

Mice pups were genotyped by polymerase chain reaction (PCR) analysis. Both genotypes (transgenic and wild-type) were used in these experiments. The mice were allowed free access to food and tap water ad libitum.

### 4.2. Cell Culture

SH-SY5Y cells (ATCC, CRL-2266) were cultured at 37 °C and 5% CO_2_ in Dulbecco’s modified eagle’s medium (DMEM; Gibco, Dublin, Ireland, 52100) supplemented with 3.7 g/L of sodium bicarbonate (Merck-Sigma Aldrich, Darmstadt, Germany, 106329), 110 mg/L of pyruvate (Merck, 106619), 2 mM of glutamine (Merck, 100289), 10% fetal bovine serum (FBS; F7524 Merck-Sigma-Aldrich, Germany), 0.01% streptomycin (PanReac AppliChem, A1852), and 100 U/mL of penicillin G (PanReac AppliChem, Darmstadt, Germany, A1837).

Primary cerebellar granule neuron (CGN) cultures were prepared as previously described [21]. Briefly, APP/PSEN1 or wild-type mouse pups were genotyped at postnatal day 2–3 (P2, P3) to perform the dissection and neuronal culture at postnatal day 5, 6 or 7 (P5–7). Primary CGNs were isolated and seeded in Neurobasal (Gibco, Ireland, 21103-049) supplemented with 1x B27 (Gibco, Ireland, 17504-044), 2 mM of GlutaMAX™ (Gibco, Dublin, Ireland, 35050-038), 0.01% streptomycin, 100 U/mL of penicillin G, and 22 mM of KCl in plates previously coated with 10 µg/mL of poly-l-lysine (Merck-Sigma-Aldrich, Darmstadt, Germany, P4707). There were 1.5 × 10^6^ cells per well, plated in 6-well plates for western blot, and 10^5^ cells per well in 24-well plates for immunofluorescence assays or to measure the secreted amyloid levels in the media by ELISA. The CGN cultures were incubated at 37 °C and 5% CO_2_, and experiments were performed until DIV 4 (4 days in vitro) in a serum-free medium to prevent the consecutive boost of glial cells (in our hands: 96% neurons and 4% glia).

Both CGNs and SH-SY5Y cells were treated with trehalose (Treh; D-(+)-trehalose dehydrate, amsbio) dissolved in MilliQ water. Rapamycin (Rapa; LC Laboratories, Woburn MA, USA, R-5000), bafilomycin A1 (BafA1; Santa Cruz Biotechnology, Santa Cruz, CA, USA, sc-201550), VPS34-IN1 (IN1; MRC Protein Phosphorylation and Ubiquitylation Unit, VPS34-IN1 [32]) and MRT68921 (MRT; MRT0068921 was kindly provided by Dr. Barbara Saxty, MRC Technology [34]) were used. The solvent dimethyl sulfoxide (DMSO, PanReac AppliChem, Darmstadt, Germany, A3672) was employed as a negative control.

### 4.3. Autophagic Flux

To measure autophagic flux, autophagosome-lysosome fusion and clearance was blocked by addition of the v-ATPase inhibitor BafA1 (or equal volume of DMSO as the control) for the last 4 h before harvesting the cells. LC3-II levels were measured by western blot on methanol-activated PVDF (Merck-Millipore, Darmstadt Germany) membranes.

### 4.4. Gel Electrophoresis and Western Blots

Cultured cells were scraped off of the plates in 100 µL of SDS-lysis buffer (50 mM of Tris pH 7.6, 400 mM of NaCl, 1 mM of EDTA, 1 mM of EGTA, and 1% SDS), heated at 95 °C with vigorous shaking for 15 min and sonicated for 30 s. Samples were centrifuged at 16,000× *g* for 20 min and the supernatants were stored at −20 °C until use.

The DC protein assay (Bio-Rad, Madrid, Spain, 5000111) was used to determine the protein concentrations. Protein samples were mixed with 5x loading buffer (10% SDS, 5% beta-mercaptoethanol, 325 mM of TrisHCl pH 6.8, 25% glycerol, and 0.5% bromophenol blue) to a final concentration of 1x, and heated for 5 min at 100 °C. Samples were resolved by SDS/PAGE and transferred to nitrocellulose (Whatman, Maidstone, KT, UK) or PVDF (Merck-Millipore, Darmstadt, Germany) membranes. After blocking with 5% BSA for 1 h, the membranes were incubated overnight at 4 °C with the primary antibody and with a secondary horseradish peroxidase-conjugated antibody for 45 min. The washing steps and solutions were made in 0.1% Tween 20 (Merck, 822184) –TBS (100 mM of Tris pH 8, and 150 mM of NaCl). Western LightningTM Plus ECL (Perkin-Elmer, Waltham, MA, USA, NEL105) was employed to detect antibody binding. GAPDH or ACTB/β-actin were used as an internal control. ImageJ software was used to measure the relative quantity of protein levels in the western blots.

The following antibodies and dilutions were used: APP-6E10 (1:1000; Covance, Princeton, NJ, USA, SIG-39300), APP C-terminal (1:1000; Sigma, A8717), BACE1 (1:1000; Cell Signaling Technology, Danvers, MA, USA 5606), cleaved CASP3 (1:1000; Cell Signaling Technology, Danvers, MA, USA, 9661), LC3B (1:4000; Sigma-Aldrich, Darmstadt, Germany, L7543), NBR1 (1:500; Santa Cruz Biotechnology, Dallas, TX, USA, sc-130380), SQSTM1 (1:4000; Novus Biologicals, UK, H00008878-M01), p-RPS6KB1 T389 (1:1000; Cell Signaling Technology, Danvers, MA, USA, 9205), RPS6KB1α (1:500; Santa Cruz Biotechnology, Santa Cruz, CA, USA sc-230), p-RPS6 S240/244 (1:1000; Cell Signaling Technology, Danvers, MA, USA, 2215), LAMP1 (1:1000; DSHB, 1D4B), GAPDH (1:2000; Cell Signaling Technology, Danvers, MA, USA, 2118), Rab 7 (1:100, Santa Cruz Biotechnology, Dallas, TX, USA, sc-376362 ACTB (1:40,000; Sigma-Aldrich, Darmstadt, Germany, A5441), anti-rabbit IgG-HRP (1:5000; Santa Cruz Biotechnology, Santa Cruz, CA, USA, sc-2004), and anti-mouse IgG-HRP (1:5000; Santa Cruz Biotechnology, CA, USA, sc-2005).

### 4.5. Aβ Quantification by ELISA 

The secreted Aβ levels of cultured neurons were measured using the Human Aβ40 Kit (Invitrogen™, ThermoFisher, Burlington, MA, USA, KHB3482) following the manufacturer’s instructions. For the intracellular determination of Aβ40, cultured CGNs were scraped off of the plates in 0.5% Triton X-100 and 2.5 mM of EDTA in PBS, incubated for 30 min at 4 °C, sonicated for 30 s, and centrifuged at 16,400× *g* for 10 min at 4 °C. The supernatants were stored at −20 °C until use. The plate absorbance was read with an Opsys MR microplate reader (Dynex Technologies, Chantilly, VA, USA) at 450 nm [44].

### 4.6. Immunofluorescence

CGNs were cultured on glass coverslips pre-treated with poly-l-lysine and fixed with 4% paraformaldehyde (PFA; Sigma-Aldrich, Darmstadt, Germany, 16005) in PBS. A 1 h incubation in PGT solution (PBS1x, 0.22% gelatin from cold water fish skin (Sigma), and 0.1% Triton X-100 (Merck, Darmstadt, Germany 108603)) was used for simultaneous blocking and permeabilization. Primary antibodies were incubated in PGT overnight at 4 °C: APP A4-22C11 (1:500; Merck-Millipore, Darmstadt, Germany, MAB348), BACE1 (1:100; Cell Signaling Tech., Danvers, MA, USA, 5606), LAMP1 (1:100; DSHB, 1D4B), and TUJ1 (beta3 tubulin; 1:1000; Santa Cruz Biotech., Santa Cruz, CA, USA, sc-58888). Alexa Fluor secondary antibodies (1:1000; Molecular Probes, Invitrogen, Burlington, MA, USA) were incubated in PBS for 1 h, followed by 10 min with DAPI (2-(4-amidinophenyl)-1H-indole-6-carboxamidine; Calbiochem, Burlington, MA, USA) to visualize the nuclei. Abundant washing with PBS was performed between steps. The coverslips were mounted with Fluoromount G (Southern Biotechnology Associates, Inc., Birmingham, AL, USA). Fluorescence signal was observed in a laser scanning confocal microscope LSM710 coupled with an upright Axio Imager.M2 (Zeiss, Jena, Germany). The images were analyzed with ImageJ software.

For colocalization analysis, the intensity correlation analysis (ICA) algorithm was employed to determine the intensity correlation quotient (ICQ) in ImageJ.

### 4.7. Aβ Endocytosis Assay

The CGN cultures from wild-type mice were treated for 4 h in the presence of 2 µg/mL of HiLyte™ Fluor 555 labeled-Aβ (1-40) (Aβ555, AnaSpec, Inc., Fremont, CA, USA). After several washes with PBS to remove non-attached Aβ555, cells were fixed with PFA and the immunofluorescence protocol was performed. TUJ1 was employed to define neuronal areas and only quantify the internalized Aβ555 using a laser scanning confocal microscope LSM510 coupled to an upright Axio Imager.Z1 M (Zeiss). ImageJ was used to quantify the total intensity of internalized Aβ555 related to neuronal area (IntDen Aβ555/Area TUJ1).

### 4.8. LysoSensor Assay

CGNs were plated in glass-bottom dishes (MatTek, Bratislava, Slovak, P35G-1.5-10-C). During the last hour of treatment, CGNs were incubated with 1 µM of the acidotropic probe LysoSensor™ Green DND-189 (Molecular Probes) for 1 h at 37 °C and 5% CO_2_. The non-internalized probe was washed with supplemented Neurobasal, and images were immediately taken in vivo using an inverted microscope Axiovert 200 (Zeiss) coupled with a monochrome sCMOS camera.

### 4.9. Cathepsin D Activity Assay

Cultured CGNs were scraped off of the plates in 50 mM sodium acetate pH 5.5, 0.1 M NaCl, 1 mM EDTA, 0.2% Triton X-100, and incubated for 1 h at 4 °C. A total of 30 µg of each sample were incubated with 10 µM fluorogenic cathepsin D & E substrate (BML-P145; Enzo Life Sciences, Farmingdale, NY, USA) at 37 °C for 30 min. The fluorescence intensity was determined in an Infinite F200 (TECAN) plate reader. An E64d (BML-PI107; Enzo Life Sciences) is a broad-spectrum cell-permeable inhibitor of cysteine proteases, which was used (2 µg/mL) to prevent the additional activation of cathepsin D by cysteine proteases during incubation with the fluorogenic substrate [37,38,48,49].

### 4.10. Statistical Analysis

Student’s t-tests or ANOVA, followed by a Holm–Sidak post hoc test, were performed with SigmaPlot software (London, UK) according to the number of variables, experimental groups, and the data distribution. Differences were considered statistically significant when *p* ≤ 0.05. All experiments were independently replicated three times, unless otherwise specified in the legend. The western blot and ELISA data are represented as mean ± standard error of the mean (SEM) on the bar charts. Data from the immunofluorescence study are represented as box plots, which show the 25th, 50th, and 75th percentiles as boxes, the 10th and 90th percentiles as error bars, and the outliers as dots.

## 5. Conclusions

Here we show an autophagy-independent anti-amyloidogenic effect of trehalose in neurons from an AD mouse model. While trehalose was able to induce autophagy in neurons only in the short-term, in the long-term trehalose provoked alterations in the endomembrane system without affecting the degradation ability of the autophagy-lysosome pathway. Interestingly, this long-term effect of trehalose reduced the colocalization of APP and BACE1, preventing the β-secretase from initiating the amyloidogenic cascade, and therefore lowering the generation of Aβ peptide. This result, together with the fact that trehalose is not degraded by mammalian cells and is able to cross the blood-brain barrier, supports the continuing relevance of research on the therapeutic potential of trehalose against AD.

## Data Availability

All data is available in manuscript and Supplementary Material.

## Supplementary Materials

The following are available online at https://www.mdpi.com/article/10.3390/metabo11070421/s1, Figure S1: Time-dependent effect of trehalose on autophagy, Figure S2: Determination of the differential neuronal autophagic flux induced by trehalose, Figure S3: IN1 and MRT prevent the increase of LC3-II levels observed with trehalose, Figure S4: Trehalose treatment provokes alterations in the endomembrane system, Figure S5: Trehalose does not modify the localization of APP or BACE1 to the lysosomes.

## Abbreviations

Aβ: β-amyloid; ACTB: beta-actin; AD: Alzheimer disease; APP: Aβ precursor protein; APP/PSEN1: B6.Cg-Tg (APPSwe, PSEN1dE9) 85Dbo/J; ATG: autophagy related; BafA1: bafilomycin A1; BACE1: beta-secretase 1; CGN: cerebellar granule neuron; GAPDH: glyceraldehyde-3-phosphate dehydrogenase; IN1: PIK3C3/VPS34-IN1; LC3: microtubule associated protein 1 light chain 3; MRT: MRT68921; MTORC1: mechanistic target of rapamycin kinase complex 1; NBR1: neighbour of Brca1 gene; PI3KCIII: the phospha-tidylinositol 3-kinase class III; Rapa: rapamycin; RPS6KB1/S6K: ribosomal protein S6 (RPS6) kinase polypeptide 1; SQSTM1/p62: sequestosome 1; Treh: trehalose; ULK: unc-51 like autophagy activating kinase; and WT: wild type.
