# Supplementary material for: Trehalose Reduces the Secreted Beta-Amyloid Levels in Primary Neurons Independently of Autophagy Induction
Source: Metabolites. 2021 Jun 26;11(7):421. doi: 10.3390/metabo11070421 (PMC8306653; doi:10.3390/metabo11070421)
Supplement: Supplementary file 1 [file metabolites-11-00421-s001.zip › metabolites-1274322-supplementary.pdf]

Supplementary information

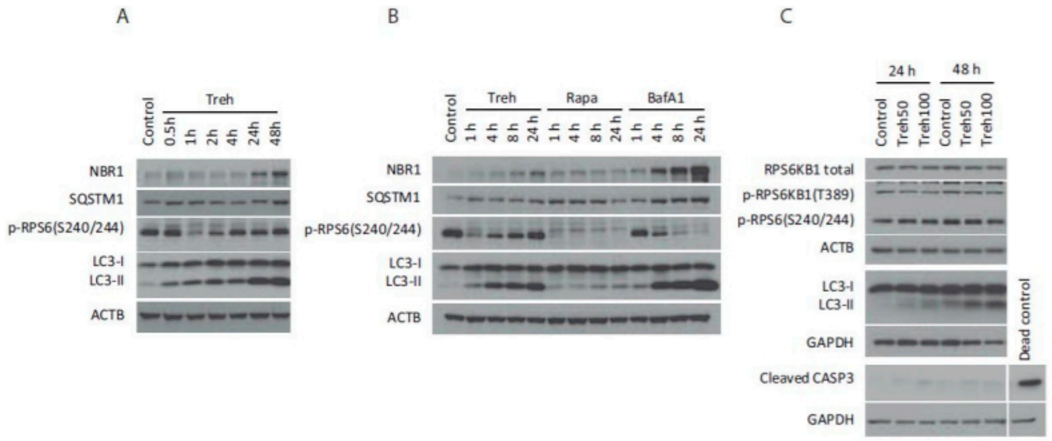

Figure S1. Time-dependent effect of trehalose on autophagy. A-B) Western blot analysis of SH-SY5Y cultures treated with 100 mM Treh, 200 nM rapamycin (Rapa) or 100 nM bafilomycin A1 (BafA1) for the indicated periods of time. C) Western blot analysis of CGN cultures treated with 50 mM or 100 mM trehalose (Treh) for 24 h or 48h. NBR1, SQSTM1 and LC3-II were measured to determine the autophagic status, p-RPS6KB1 T389 and p-RPS6 S240/244 as indicators of MTORC1 activity, cleaved CASP3 to determine the cellular viability and ACTB or GAPDH as internal controls.

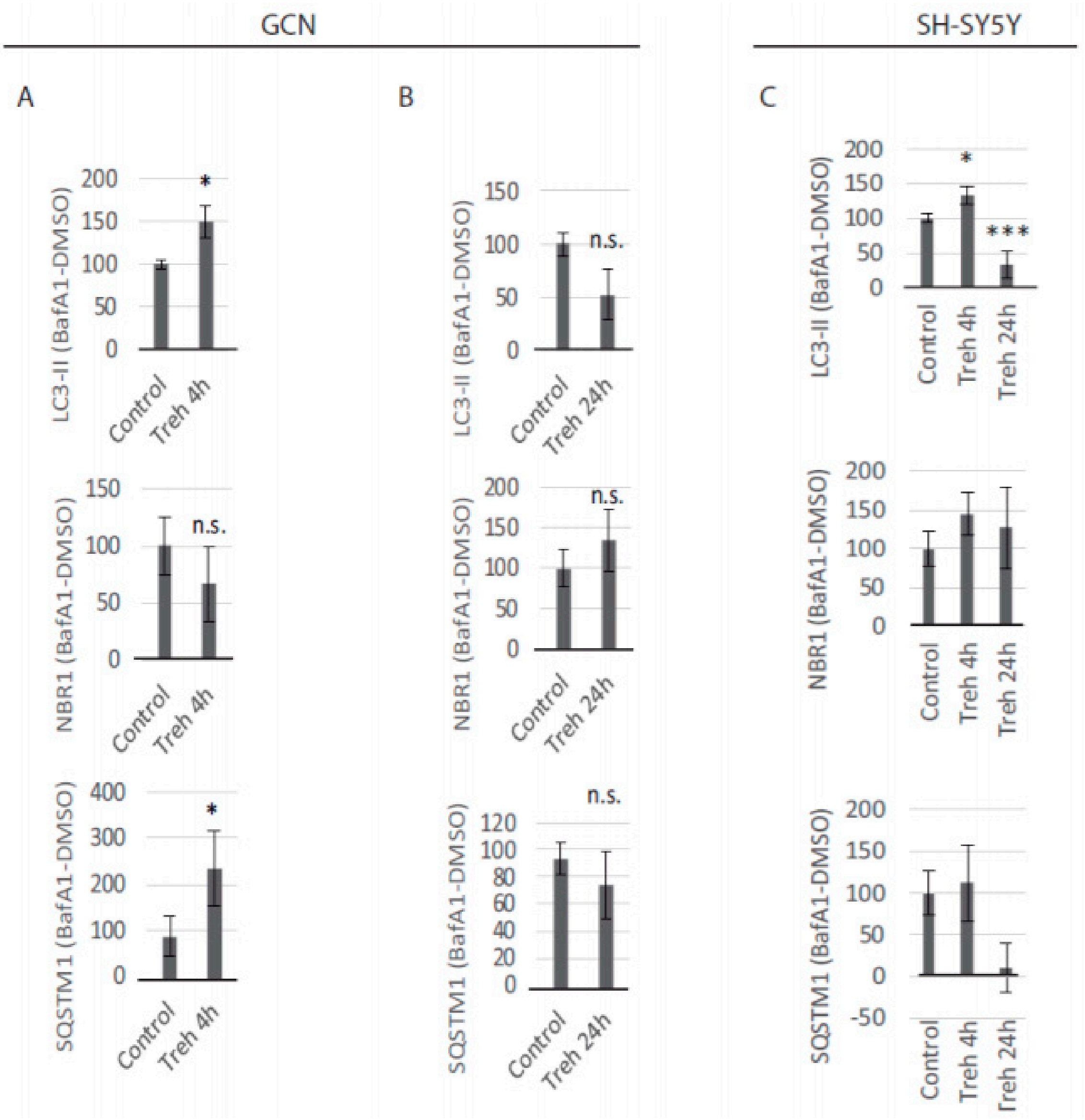

Figure S2. Determination of the differential neuronal autophagic flux induced by trehalose. Autophagic flux analysis was determined by the addition of 100 nM bafilomycin A1 (BafA1) or DMSO as vehicle, during the last 4 h of treatment with 100 mM Treh for 4 h (A) or 24h (B) in CGN cultures; or in SH-SY5Y neuroblastoma for 4 h and 24 h (C). The quantitative data BafA1 versus Control were normalized and represented as 100 relative units. Student's t-test was performed for comparisons of NBR1, SQSTM1 and LC3-II levels (n ≥ 3; * p ≤ 0.05; ** p ≤ 0.01; *** p ≤ 0.001). Bars represent mean ± SEM.

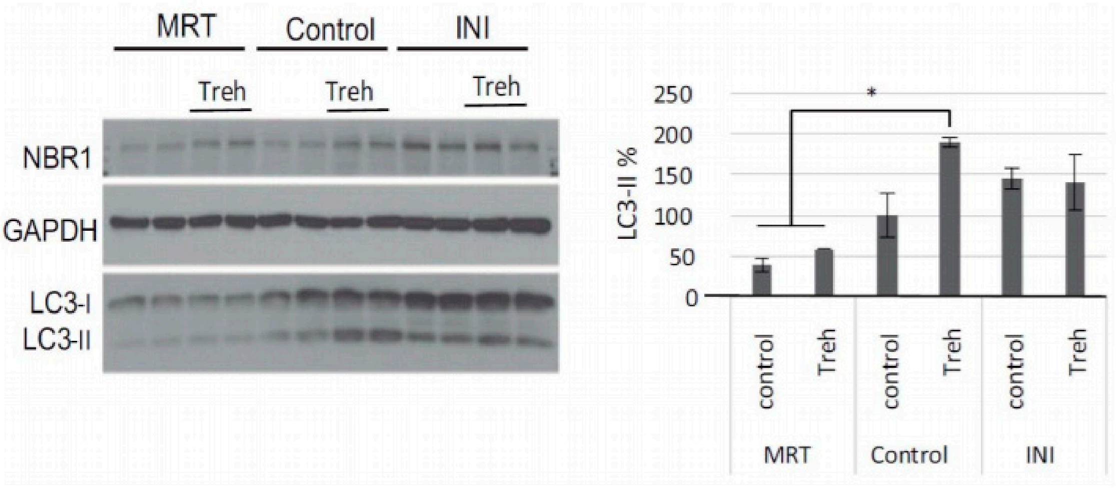

Figure S3. IN1 and MRT prevent the increase of LC3-II levels observed with trehalose. CGN cultures were treated with 100 mM Treh in the presence/absence of the inhibitors of autophagosome formation PIK3C3/VPS34-inhibitor 1 (IN1) or MRT68921 (MRT). Western blot analysis of the autophagic markers LC3-II and NBR1 from cellular extracts after 24 h of treatment with 1 μM IN1 or 1 μM MRT. Two-way ANOVA (n = 2); GAPDH used as internal control.

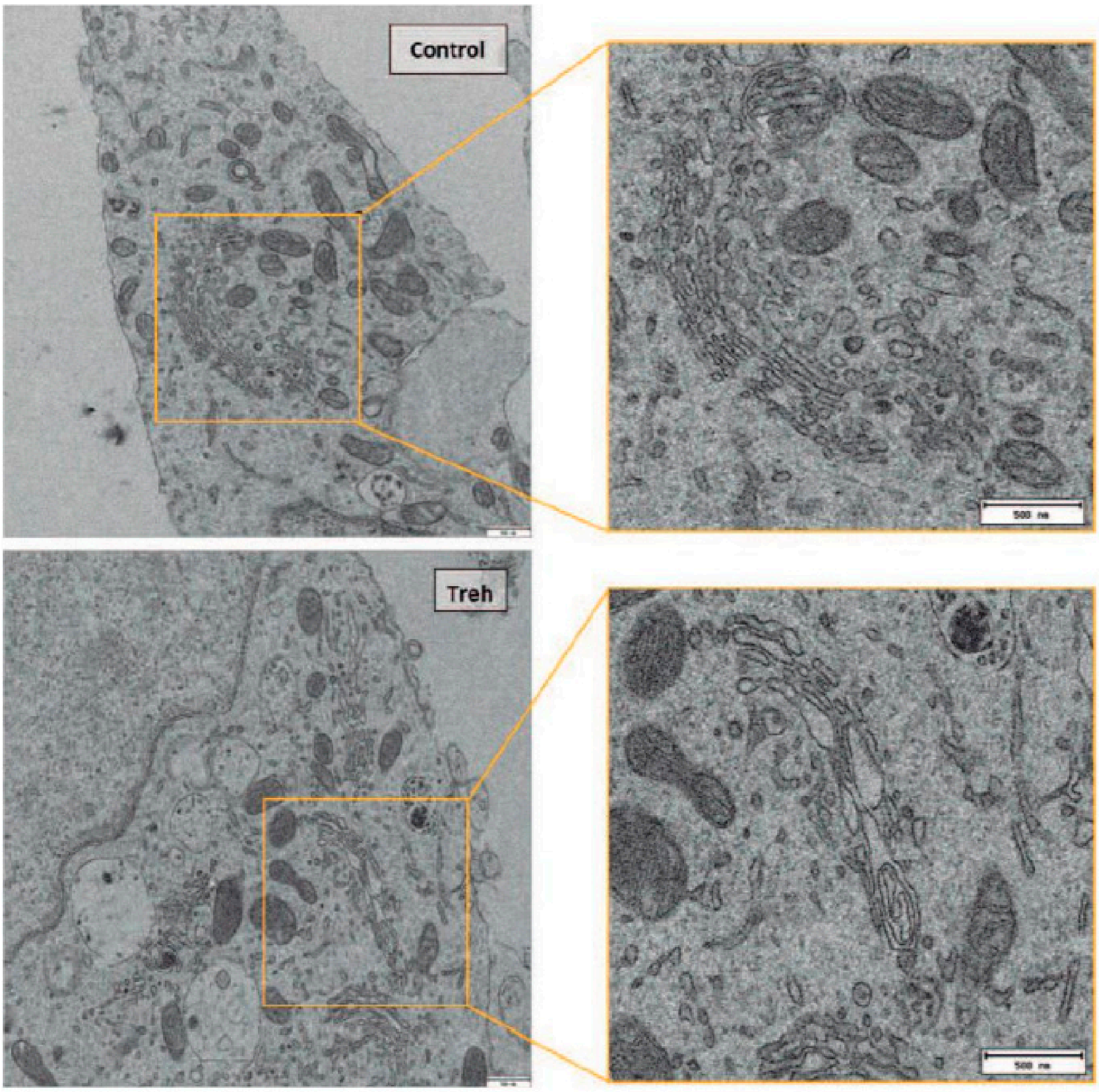

Figure S4. Trehalose treatment provokes alterations in the endomembrane system. CGN cultures were treated with 100 mM Treh for 24 h and processed for its analysis by transmission electron microscopy. Scale bar: 500 nM.

Electron microscopy methods: CGN cultures were prepared (4 x 106 cells per P60), treated and fixed with PFA 4% + 2% glutaraldehyde in Sörensen buffer (Na/K 0.1 M pH 7.4), which was kept 2 h at RT. Sample preparation was carried out by the electron microscopy service of the CBMSO. Briefly, after washing, the cells were centrifuged for 5 min at 2000 x g. A post fixation with 1% osmium tetroxide and 1% potassium ferricyanide was carried out for 1 h at 4 ºC. After three washes in double distilled water, 0.15% tannic acid in Sörensen buffer was added for 1 min. After several washes, it was incubated with 2% uranyl acetate 1 h at 4 °C in the dark. After three washes with double distilled water, the samples were dehydrated at 4 ºC with increasing concentrations of acetone (50, 70, 90 and 100%) for 10 min per incubation. For infiltration into the Epon type epoxy resin (TAAB 812, TAAB laboratories, England), it was incubated at RT while shaking in a mixture of acetone and Epon resin 3:1 for 1 h and then 1:1 overnight. The next day it was incubated in acetone-Epon 1:3 1 h, Epon 100% was

renewed at 6 h and 24 h. Samples were polymerized at 60 °C for 2 days. Ultrafine 70-nm slices were obtained on an ULTRACUT UCT ultramicrotome (Leica, Vienna) with a DIATOME diamond blade. The sections were collected on Cu/Pd (100 mesh) grids covered with Formvar (TAAB laboratories, England) and a layer of evaporated carbon. The sections were stained with 2% uranyl acetate for 7 min and with Reynolds lead citrate for 2 min. We obtained the images with a Jeol JEM-1010 electron microscope (Jeol, Japan) with an electron acceleration voltage of 80 kV, and a 4Kx4K CMOS camera, F416 from TVIPS (Gauting, Germany).

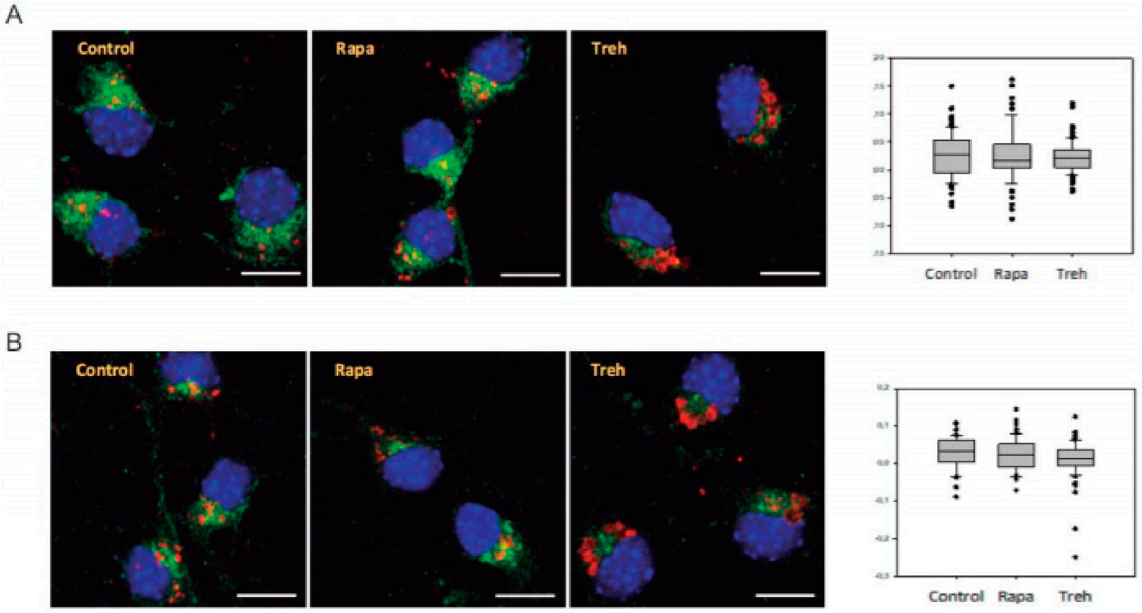

Figure S5. Trehalose does not modify the localization of APP or BACE1 to the lysosomes. CGN cultures were treated for 24 h with 200 nM rapamycin (Rapa) or 100 mM trehalose (Treh). Colocalization analysis of A) APP (22C11, green) and LAMP1 (red) or B) BACE1 (green) and LAMP1 (red). Images were analyzed with ImageJ software. Intensity Correlation Analysis (ICA) algorithm was employed to determine the Intensity Correlation Quotient (ICQ), with no significant differences observed. Nuclei were stained with DAPI; scale bar: 10 µm. 70 neurons were quantified per experimental condition. Box plots represent 10th, 25th, 50th, 75th and 90th percentiles as boxes and error bars, and outliers as dots.
